# Effect of Low-Temperature-High-Pressure Treatment on the Reduction of *Escherichia coli* in Milk

**DOI:** 10.3390/foods9121742

**Published:** 2020-11-26

**Authors:** Yifan Li, Zhuoyun Zheng, Songming Zhu, Hosahalli S. Ramaswamy, Yong Yu

**Affiliations:** 1 College of Biosystems Engineering and Food Science, Zhejiang University, 866 Yuhangtang Road, Hangzhou 310058, China; yifanli@zju.edu.cn (Y.L.); 21813047@zju.edu.cn (Z.Z.); zhusm@zju.edu.cn (S.Z.); 2 Key Laboratory of Equipment and Informatization in Environment Controlled Agriculture, Ministry of Agriculture, 866 Yuhangtang Road, Hangzhou 310058, China; 3 Department of Food Science, McGill University, 21111 Lakeshore Road, St-Anne-de-Bellevue, QC H9X 3V9, Canada; Hosahalli.Ramaswamy@Mcgill.Ca

**Keywords:** low-temperature-high-pressure (LTHP), *Escherichia coli*, milk, non-thermal processing, microbial inactivation, food safety

## Abstract

Non-thermal processing of milk can potentially reduce nutrient loss, and a low-temperature-high-pressure (LTHP) treatment is considered as a promising alternative to thermal treatment, attracting considerable attention in recent years. The effect of LTHP treatment (−25 °C, 100–400 MPa) on the phase transition behavior of frozen milk was evaluated. The lethal and injured effects of different pressures and cycle numbers on *E. coli* in frozen milk were studied by using selective and non-selective enumeration media. Results from the gathered transient time–temperature–pressure data showed that pressures over 300 MPa could induce a phase transition from Ice I to Ice III. The treatment at −25 °C and 300 MPa could achieve a lethal effect similar to the two-cycle treatment of 400 MPa at room temperature. This meant that LTHP conditions can lower the operating pressure by at least 100 MPa or reduce the operation from two cycle to one cycle. Increasing the number of pressure cycles enhanced the lethal effects, which was not additive, but resulted in a transformation of part of the injured cells into dead cells. Transmission electron microscopy (TEM) and scanning electron microscopy (SEM) provided direct evidence for the breakdown of cell membrane and cell walls by phase transitions. Combined with a designed internal cooling device, the LTHP process can be expected to be a more attractive alternative to non-thermal processing for the dairy industry.

## 1. Introduction

Milk is highly perishable and can harbor a number of microorganisms that are of spoilage and safety concern and can become an important source of foodborne pathogens, and inadequate pasteurization/sterilization treatments may cause safety issues for consumers [1]. In order to ensure food safety, most countries require that dairy products must undergo appropriate thermal pasteurization or commercial sterilization (e.g., 72 °C for 15 s, 105–120 °C for 20–40 min, or 135–150 °C for 1–4 s) [2]. Thermal processing is considered to be the most effective way to destroy milk pathogens, but it has a negative effect on the resulting quality of the milk. For example, milk proteins may denature during heating, causing them to lose their quaternary and tertiary structures and form unfolded random shapes. The vitamins present in milk may get degraded. Non-thermal technologies can also be used to inactivate milk pathogens but result in better retention of nutrients. Therefore, the development of new non-thermal processing technologies is of great importance to consumers and the food industry as a whole [3,4,5].

High-pressure processing (HPP) is a purely physical, non-thermal processing method, which is considered as a promising alternative to heat treatment for pasteurization and sterilization, but there are only few reports demonstrating the commercial success of HPP milk [6]. Studies have confirmed that HPP could be a convenient alternative to produce microbiologically safe milk with fresh-like characteristics. In fact, much effort has been focused on the HPP of milk for pasteurization and refrigerated storage, but HPP sterilization is rather limited because of the difficulty in destroying spore-forming microorganisms, which may easily cause milk spoilage [7,8]. A number of studies have demonstrated that increasing the pressure, extending the holding time, employing a higher temperature, and the combined effect of multiple factors (e.g., combing high pressure (HP) with nisin and heat) can enhance the sterilization action [7,9,10,11]. Some pathogenic bacteria, especially the pressure-resistant pathogens, need to be inactivated at the pressure levels above 600 MPa [12]. Furthermore, under lower operating pressures, HPP requires relatively long holding times (>30 min) to inactivate microorganisms [11]. Obviously, these methods have not been commercially viable because they are inefficient; the process has high capital and maintenance costs, increases wear and tear, causes a shortened equipment life, and has a lower productivity [13]. Processing conditions, such as 350 MPa for 120 min or 400 MPa for 30 min, will never be commercially used to inactivate microorganisms in food, as one of the most important requirements of a commercial process is reduced processing time [14].

It has been observed that, like many novel methods of food preservation, HP treatment of milk may only cause damage to some cells, not completely killing them; such damaged cells could eventually recover and multiply during the storage period. In such cases, even the damaged ones will be considered as killed because the selective media employed for some bacteria may not permit the growth of injured cells and therefore even the injured ones will be taken as killed; thus, overestimating the destruction rate [15,16,17]. Because of the potential recovery from this storage process, a 400–600 MPa treatment can only cause sub-lethal injury to *E. coli*, thus compromising food safety [16]. Studies have shown that the lethality and injury of HP-treated microorganisms can be detected by selective and non-selective agar mediums [15,17]. On the other hand, sub-zero temperatures can have a protective effect on the quality of dairy products, especially for cheese and curds. Fava et al. [18] reported that milk under cooling (5 °C) and freezing (−5 °C) for seven days had no significant difference, and short-term freezing did not affect the chemical composition or physical properties of milk. The study of Bottiroli et al. [19] also indicated that short-term freezing at −20 °C and −80 °C could be used for milk storage without changing the sensory properties or the physical stability of milk.

The low-temperature-high-pressure (LTHP) method is a non-thermal processing technology that has attracted considerable attention in recent years; it implies a high-pressure treatment carried out at lower temperatures in general, but more specifically, even at subzero (frozen) temperatures, as employed in this study. By controlling different temperature and pressure conditions, ice can form different structures (e.g., Ice I, Ice II, Ice III, and Ice V) [20]. Ice I is the common ice structure at atmospheric pressure; its density is less than water, while other structures are denser than water [21]. Test samples are made to undergo Ice I to Ice III phase transitions induced by freezing temperatures and elevated pressures. The strengthening effect of its lethality has been verified [22]. The study of Shen et al. [23] reported that after Ice I to Ice III phase transitions, treatment time had limited influence on the loss of viability of *Bacillus subtilis* treated by LTHP (no significant correlation), indicating that the lethal effect depended primarily on the associated phase transitions. The specific lethal effects and mechanisms of LTHP are still controversial, and the accepted explanations include damages of the cell membrane, membrane potential changes induced by osmotic active substances (e.g., Na^+^ and K^+^), inactivation of crucial intracellular enzymes, and so on [6,23,24,25].

Available LTHP methods used in the literature reports employ externally supplied refrigeration systems to cool the pressure chamber to subzero temperatures [23,26], resulting in not only slow cooling rates, but also complicating the design of the equipment. Schlüter et al. [27] designed a high-pressure vessel specifically for LTHP processing. The vessel had an inner volume of 3.7 mL and a maximum design pressure of 1.0 GPa in the temperature range of −50 to +150 °C. Silicone oil was used as the pressure-transmitting medium. The LTHP processing was achieved by immersing the vessel in a cryostat. Our team previously reported that, at the sub-lethal levels, the pressure sterilization effects were enhanced at lower temperatures [28]; we also designed a simple approach to carry out such studies through a specially designed modification of the HP applicator. A new design of an insulated container was developed and successfully demonstrated to be able to induce ice phase transition from Ice I to Ice III in frozen samples. The container permits to freeze the samples inside the pressure chamber, without the need for an external cooling system, and hence regular HP equipment could be used for the treatment. The lethal effects under higher pressures could be realized by using the designed container for studying destruction kinetics under lower temperatures and higher pressures, thus greatly improving the cooling rate and reducing equipment wear [29,30].

The objective of this study was to evaluate the phase transition behavior of inoculated milk during LTHP treatment. The previously designed container was used to realize the rapid cooling and phase transitions of milk inside the pressure chamber. The lethal and injuring effects of the LTHP treatment on *E. coli* were studied by selective and non-selective agar media. The lethal mechanisms towards *E. coli* were preliminarily investigated by using SEM and TEM, to provide a theoretical basis for the commercial application of LTHP treatments.

## 2. Materials and Methods

### 2.1. Bacterial Culture Preparation

*Escherichia coli* ATCC 25,922 (CGMCC 1.2385) culture was obtained from the China General Microbiological Culture Collection Center (CGMCC, Beijing, China), plated on nutrient agar (Beijing Solarbio Science & Technology Co., Ltd., Beijing, China) every 2 weeks to maintain its activity. Prior to each experiment, several loops of stock culture were inoculated into 50 mL nutrient broth (Shanghai Acmec Biochemical Co., Ltd., Shanghai, China) and incubated in a thermostatic oscillator bath (HZ-9211KB, Taicang Hualida Experimental Instrument Co., Ltd., Suzhou, China) at 37 °C for 24 h with mild agitation (150 rpm). Several loops of the *E. coli* suspension broth were re-inoculated and cultured as above in a biochemical incubator (SPX-450, Ningbo Saifu Experimental Instrument Co., Ltd., Ningbo, China) at 37 °C for 24 h.

### 2.2. Preparation of E. coli-Inoculated Milk

The fresh, whole milk used in this study was purchased from Zhejiang Inm Food Co., Ltd., Wenzhou, China, with a 5% protein content and 6% fat content. The original fat content was maintained although it is recognized that it can increase the resistance to thermal destruction of *E. coli* in milk [31] in order to obtain the worst-case scenario. The fresh milk had a pH of 6.65 (FE-20, Mettler-Toledo Instruments Co., Ltd., Shanghai, China). Before the experiment, the milk was put into a thermostatic water bath (N3–8, Hangzhou Bioer Technology Co. Ltd., Hangzhou, China) and pasteurized at 65 °C for 30 min in order to kill the vegetative bacteria present in the milk. A total of 30 mL of the incubated broth above was transferred into a 50 mL sterilized centrifuge tube and centrifuged at 3200 *g* for 5 min at 20 °C (5810R, Eppendorf AG, Hamburg, Germany). The obtained *E. coli* pellet was washed in 0.5 M potassium dihydrogen phosphate buffer (pH 7.2, Sinopharm Chemical Reagent Co., Ltd., Shanghai, China) and re-suspended in 30 mL pasteurized milk. The initial population of *E. coli* in the inoculated pasteurized milk was about 10^9^ CFU/mL. The inoculated pasteurized milk was transferred into 9 mL cryogenic vials for subsequent experiments.

### 2.3. Insulated Container

**Figure 1:**
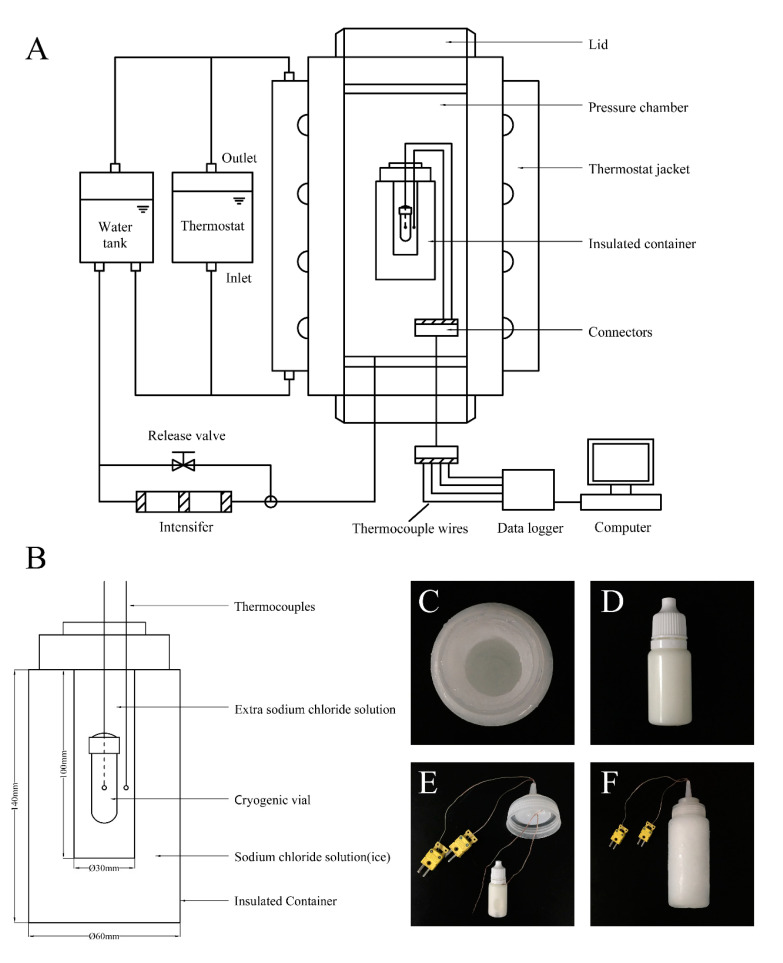
(**A**) Schematic diagram of the experimental high-pressure apparatus. (**B**) Schematic cross-section diagram of the insulated container. (**C**) The ice hole created by pulling out the centrifuge tube after freezing. (**D**) The cryogenic vial filled with inoculated pasteurized milk. (**E**) The thermocouples inserted into the plastic cap and cryogenic vial. (**F**) The screwed-on insulated container.

The insulated container fabricated and evaluated in the previous study [28] was used and some further modifications were made in order to be more suitable for this study. A 300 mL plastic bottle was filled with a sodium chloride solution (20%, *w*/*w*) and a 50 mL centrifuge tube was glued onto the cap, and then screwed into the body to extrude the overflow water. The plastic bottle was frozen at −25 °C for 48 h, creating an ice hole with a diameter of 30 mm and a depth of 100 mm when the centrifuge tube was pulling out of the 300 mL bottle. This ice hole ensured that prepared cryogenic vials were placed in the center of the insulated container and reduced the freezing time of the sample. Two K-type thermocouples (OMEGA Engineering, Stamford, CT, USA) were passed through the plastic cap for temperature recording using a data logger (34970A, Agilent Technologies Inc., Santa Clara, CA, USA). One was inserted into the cryogenic vial to measure the core temperature of the sample and the other was used to measure the temperature around the cryogenic vial. After the thermocouples were installed, the cryogenic vial was placed in the ice hole, filled with a sodium chloride solution (20%, *w*/*w*), and the cap was screwed on (details shown in Figure 1). The insulated container was vacuum-packed in a flexible thermo-stable PA/PE pouch and then frozen at −25 °C for 24 h.

### 2.4. High-Pressure Treatment

As shown in Figure 1A, a laboratory-scale high pressure equipment was used for the HP treatments, with a maximum chamber capacity of 5 L (UHPF-750, Baotou Kefa High Pressure Technology Co., Ltd., Baotou, China). The frozen samples were treated at selected pressure levels (100, 200, 300, and 400 MPa), while the unfrozen samples were only treated at the pressure of 400 MPa without refrigeration. The pressure was immediately released once the selected pressure was reached (after the come-up period). One-cycle treatment started from the beginning of the pressure increase to the completion of the pressure release. Two-cycle treatment repeats the same pressure cycle once again. The pressure boost rate of the equipment was about 150 MPa/min, and the pressure release time was less than 5 s. Pressure and temperature data were recorded at one-second intervals. The treated samples were thawed in flowing water as soon as possible and stored at 4 °C for a maximum of 2 h before microbial enumeration. Each experiment was performed at least in triplicate.

### 2.5. Enumeration of E. coli

The thawed samples were serially diluted in 0.5 M potassium dihydrogen phosphate buffer (pH 7.2). The samples were enumerated in brain–heart infusion agar (BHIA, Beijing Coolaber Science & Technology Co. Ltd., Beijing, China) and violet-red bile agar (VRBA, Shanghai Acmec Biochemical Co., Ltd., Shanghai, China) to differentiate between surviving *E. coli* with and without injury [17,32]. Untreated inoculated pasteurized milk was used as the control sample and enumerated only in BHIA media. Initial counts of *E. coli* in the frozen samples were obtained to differentiate the effect of freezing on microbial reduction. The colonies formed after 48 h of incubation at 37 °C were counted. The extent of injury and lethality achieved was calculated as follows:

Extent of injury (logarithm of injured cells) = log N_B_ − log N_V_ (1)

Extent of lethality (logarithm of killed cells) = log N_O_ − log N_B_ (2)

where N_V_ is the number of *E. coli* colony recovered in the VRBA (selective media), N_B_ is the number of *E. coli* colony recovered in the BHIA (non-selective media), and N_O_ is the number of *E. coli* colony in the control sample.

### 2.6. Scanning Electron Microscopy (SEM) and Transmission Electron Microscopy (TEM) Analyses

The treated and untreated control samples for microscopic examinations were centrifuged at 3200 *g* for 5 min at 20 °C. Two batches of each sample were made, one for the SEM analysis and the other for TEM analysis. After two washes in a phosphate buffer (0.1 M, pH 7.0), the pellets were re-suspended and fixed with 2.5% glutaraldehyde in a phosphate buffer overnight. After the *E. coli* were washed three times in the phosphate buffer for 15 min, they were post-fixed with 1% O_s_O_4_ (SPI-CHEM, West Chester, PA, USA) in the phosphate buffer for 1–2 h and washed three times in the phosphate buffer (15 min per wash). The samples were then dehydrated by a graded series of ethanol (30%, 50%, 70%, 80%, 90%, and 95%) for about 15 min at each step. After that, one batch of the samples were dehydrated two times by alcohol for 20 min at each step and finally dried in a critical point dryer (HCP-2, Hitachi Co., Ltd., Tokyo, Japan). The dried samples were coated with gold-palladium in ion sputter (E-1010, Hitachi Co., Ltd., Tokyo, Japan) for 4–5 min and observed by SEM (SU-8010, Hitachi Co., Ltd., Tokyo, Japan). The other batch was dehydrated by alcohol for 20 min and then transferred to absolute acetone (Sinopharm Chemical Reagent Co., Ltd., Shanghai, China) for 20 min. The samples were placed in a 1:1 mixture of absolute acetone and the final Spurr resin mixture for 1 h at room temperature, and then transferred to a 1:3 mixture of absolute acetone and the final resin mixture for 3 h and to a final Spurr resin mixture for overnight. The samples placed in the Eppendorf tubes contained Spurr resin (SPI-CHEM, West Chester, PA, USA) and were heated at 70 °C for more than 9 h. The specimens were sectioned using an ultramicrotome (EM UC7, Leica Microsystems, Wetzlar, Germany) and the sections were stained by uranyl acetate (SPI-CHEM, West Chester, PA, USA) and alkaline lead citrate (Sinopharm Chemical Reagent Co., Ltd., Shanghai, China) for 5 to 10 min, respectively, and finally observed by TEM (H-7650, Hitachi Co., Ltd., Tokyo, Japan).

### 2.7. Statistical Analysis

All treatments were carried out in triplicate for each experimental condition and all data were expressed as means ± standard deviation (SD). The significant differences between the results were analyzed by one-way analysis of variance (ANOVA). Duncan’s test (*p* < 0.05) was used to compare the differences in average values by using SPSS software (25.0, IBM Corp., Armonk, NY, USA).

## 3. Results and Discussion

### 3.1. Temperature and Phase Transition in Frozen Milk during HP Treatment

**Figure 2:**
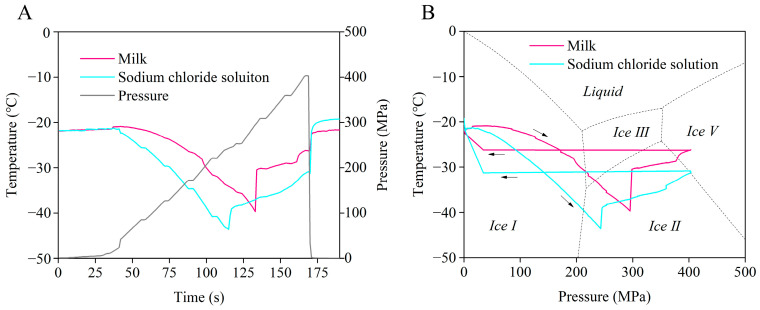
The temperature and pressure profiles of frozen milk during HP treatment: (**A**) temperature and pressure curves over time; (**B**) temperature–pressure profiles superimposed on the phase diagram of water. The black arrows indicate the direction in which the pressure increases and decreases.

Figure 2 illustrates the temperature and pressure profiles of the frozen inoculated milk during the HP-selected LTHP treatments. Due to the intermittent working principle for pressure build-up, the unit generated a stepwise ladder-like pressurization of the chamber (Figure 2A). Unlike the previous studies reported by Shen et al. [23] and Zhu et al. [29], no discontinuity (a sudden increase or decrease) of stress (at −25 °C and 400 MPa) was detected. The difference may be caused by the relative volume of the actual ice and the pressure chamber, and the frequency of the data recording. The study of Van Buggenhout et al. [33] also found no discontinuity during the LTHP treatments (−26 °C at 300 MPa, 400 MPa, and 500 MPa) of the crude enzyme extracts. Initially, the temperature of both the milk and sodium chloride solutions revealed slight increases due to pressurization and adiabatic heating (expected to be about 3 °C/100 MPa pressure increase at room temperature conditions, but much lower due to the surrounding ice heat sink). As the pressure increased, the ice formed by the sodium chloride solution outside the cryogenic vial first thawed partially, suggesting the energy requirement of pressure-induced thawing. The thawing transition was slow due to the heat transfer constraints. As a result, the temperature of the sodium chloride solution decreased along the phase transition line and approached slowly as the thawing progressed [27]. Since the heat absorbed by the outer ice crystals of the cryogenic vial exceeded the compression heating of milk, the core temperature of the milk decreased as the outer temperature decreased, helping to protect the frozen milk from thawing. This conclusion has been confirmed in previous studies as well [28,29]. Van Buggenhout et al. [34] pressurized the vessel to 200 MPa, and reported that it took about 1.5 h to cool down the sample center temperature inside the vessel from 0 to −15 °C. In our study, it took only about 1.5 min for the milk center temperature to change from −21 to −40 °C. It means that the device we designed can greatly improve the cooling rate, which is beneficial for future commercial applications.

Figure 2B shows the temperature–pressure profiles superimposed on the phase diagram of water [20]. The phase transition line of the sodium chloride solution was significantly lower than that of water (~17 °C) due to the fact that the freezing point of the sodium chloride solution is lower than that of pure water. The phase transitions of milk and the outer ice formed by the sodium chloride solution were detected at 295 MPa and 243 MPa (a sudden increase in temperature), respectively. Actually, the phase transition point of pure water is supposed to be 210 MPa [20]. This phenomenon was caused by the metastable state of Ice I in the domain of Ice Ⅲ, and different ice polymorphs have been observed in the region of Ice Ⅲ [35,36]. Shen et al. [23] summarized the pressure data for phase transitions at −25 °C and −45 °C and found that the range of transitions may vary widely (more than 100 MPa). In the meanwhile, the Ice I to Ice Ⅲ phase transition line of pure water was the average values of many data points, and in some cases, the actual measured data may vary as well. Before and after the phase transition, the temperature of the milk increased from −39.7 °C to −30.5 °C, and that of the sodium chloride solution increased from −43.6 °C to −40.5 °C. This phenomenon could actually be divided into two steps: the phase transition from the meta-stable Ice I to Ice III (endothermic reaction with an enthalpy change of 4.5 kJ/kg) and water re-crystallization to Ice III (exothermic reaction with an enthalpy change of 242.5 kJ/kg) [20,22]. The freezing point of the sodium chloride solution may not be low enough for the milk to avoid the meta-stable region in Ice III, so the milk must pass through a partial thawing process of Ice I to water, thus causing a sudden increase in temperature because of the combined effect of meta-stable Ice I to Ice III and water re-crystallization to Ice III. In contrast, the temperature decreased rapidly first (not obvious in the figure) and then increased during de-pressurization. The temperature increase was caused by the volume expansion cooling of the milk and sodium chloride solution, and the temperature increase was due to the conversions of Ice III to Ice I and water to Ice I (both exothermic reaction).

From a commercial point of view, it was unrealistic to use temperature–pressure profiles for every batch of products to determine whether phase transitions have occurred. Hence, only specific temperature and pressure parameters were supposed to be controlled to ensure the phase transition. For the purpose of this study, the temperature was determined (−25 °C) and the phase transition point of the milk was located at 300 MPa (actually 295 MPa as observed in Figure 2). This phase transition point was considered to be the critical point for re-crystallization from meta-stable Ice I to Ice III. Therefore, without changing other conditions, only the HP treatments with pressure more than 300 MPa were sufficient to induce phase transitions between Ice I and Ice III.

### 3.2. Effect of LTHP Treatment on the Lethality and Injury of E. coli in Frozen Milk

Table 1 shows the injury extent and lethality extent of *E. coli* strains in inoculated milk resulting from different treatments. After 24 h freezing at −25 °C, 0.61 log CFU/mL of injured cells and 0.75 log CFU/mL of killed cells were found. Previous studies have shown that the freezing process itself has some lethal action on microorganisms to some extent (about 1 log-reduction) [23]. Considering that freezing was also an essential part of the LTHP treatment, the initial colony of untreated (no freezing) inoculated milk was used as the control sample, meaning that all lethal levels of the *E. coli* strains were calculated based on this value.

For one-cycle treatments, there was no significant difference in lethality between the LTHP treatments at 100 MPa and 200 MPa, and the logarithms of inactivated *E. coli* were 1.33 and 1.30, respectively. When the pressure reached 300 MPa (the critical pressure of phase transition at −25 °C mentioned above), the levels of lethality doubled rapidly, increasing by more than 1.5 logarithms. The results showed that there were significant differences between the lethality at 300 MPa and 400 MPa and those at 100 MPa and 200 MPa. This is hypothesized to be caused by the phase transition from meta-stable Ice I to Ice III during the pressurization process and the re-crystallization of Ice III, or a similar phase transition during the pressure-release process. Similarly, Shen et al. [23] found that at −25 °C, inactivation of *Bacillus subtilis* cells was lower than 2 logarithms at 150 MPa but more than 4 logarithms at 250 MPa. The difference in critical points is probably due to the different strains and the media used to inoculate the microorganisms. Compared with other buffer solutions [37], more *E. coli* were able to survive in milk due to the baro-protective effect of milk on bacteria [38,39], especially the high protein and fat proportions in the market sample of whole milk [31]. For unfrozen samples, the lethal effect of one-cycle and two-cycle at 400 MPa were 2.19 log and 2.85 log, respectively. Obviously, the treatment at −25 °C and 300 MPa can achieve a lethal effect similar to the two-cycle treatment of 400 MPa at room temperature, which means that the LTHP conditions can lower the operating pressure by at least 100 MPa or reduce the operation from two cycle to one cycle. At 400 MPa LTHP, these differences were further enhanced.

For two-cycle treatments, it showed significant differences in levels of lethality before and after the 300 MPa treatment. A previous study by Su et al. [28] showed that the pressure pulse effect (PE) on unfrozen *E. coli* was well described by a linear relationship (*R*^2^ = 0.982) between the cell reductions and pressure cycle, but the PE values of frozen *E. coli* at 350 MPa and 400 MPa were highly enhanced. Zhu et al. [29] also observed significant differences in *E. coli* reductions before and after phase transition, which was similar to the results observed in this study. When the pressure was below 300 MPa, increasing pressure cycles did not enhance the lethal effect. After the phase transition occurred, continuing to increase the pressure could effectively enhance the lethal effect. These differences could also be ascribed to the fact that the microbial cells were perhaps more injured and became more sensitive to pressure after the phase transition, and the lethal effect brought by continuing to increase the same pressure might further enhance the microbial killing. In addition, the maximum pressure reached in this experiment was 400 MPa, which was actually in the range of Ice V. Considering the existence of a meta-stable state of Ice III in the domain of Ice V [22,23], although no abrupt temperature changes were observed, the possibility of Ice III to Ice V phase transition cannot be ruled out in some experiments. Van Buggenhout et al. [33] observed the Ice III to Ice V phase transition between 400 and 500 MPa. After repeated pressure cycles, meaning that the solid-to-solid phase transition of ice was acting on the sample repeatedly [22], the lethal effect was reported to enhance, but the effect of cycle number was not additive. It may be due to the pressure resistance variation of each cell in the frozen milk. When all vulnerable cells were destroyed, even increasing the number of cycles cannot bring further lethal effect [39] because only the pressure-resistant fractions may remain. Moreover, the repetition of compression and decompression phases could contribute to change the distribution of the cell resistance and to leave baro-resistant survivors [40]. However, it must be pointed out that the treatment at −25 °C and 300 MPa can achieve a sufficiently high lethal effect, and whether it is necessary to increase the pressure may depend on the lethal requirements for commercial applications.

Despite the large number of previous studies on recovery of sub-lethal injury [15,16,41], there appear to have been no previous reports about injury of LTHP-treated cells. For one-cycle pressure treatments, it could be observed that the injury effect of *E. coli* was significantly enhanced after the phase transition. Comparing different pressure cycles, the injury levels at 300 MPa and 400 MPa decreased significantly. Combined with the extent of the lethality, it could be concluded that the increase in lethal levels at these two pressures was mainly due to the transformation of injured cells into lethal cells, meaning that increasing pressure cycles may result in a more lethal effect than injury. Koseki et al. [37] verified that the increase in bacterial number during storage after HP treatment was due to the repair of injured cells rather than bacterial growth. Bozoglu et al. [15] divided bacterial injuries into primary (I1) and secondary (I2) types. Once I2 to I1 repair is completed, injured cells can rapidly become active cells. Therefore, it is very important to control the number of sub-lethal injured cells, because these cells can potentially repair themselves and grow during storage causing microbial disease [7]. Especially for low-acid foods such as milk, ignoring the recovery of foodborne pathogens may lead to overestimation of safety [42,43].

### 3.3. Microstructure Observations of Untreated and Treated E. coli

**Figure 3:**
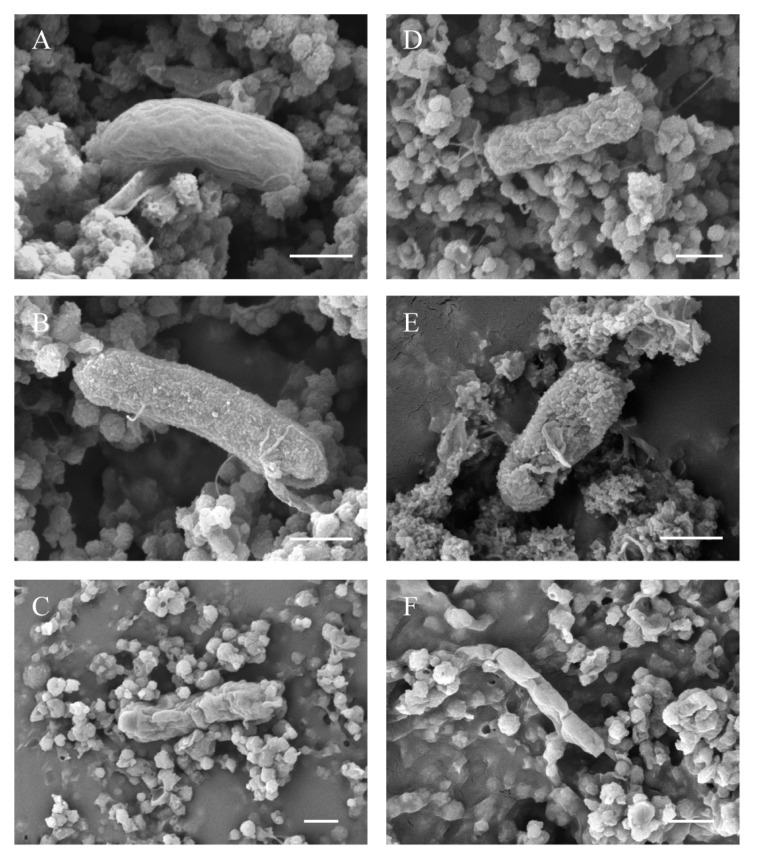
Scanning electron micrographs of *E. coli* resulting from different treatments: (**A**) control group (×40,000); (**B**) freezing (×40,000); (**C**) HP 400 MPa (×22,000); (**D**) LTHP 200 MPa (×30,000); (**E**) LTHP 400 MPa (×40,000); (**F**) LTHP two-cycle 400 MPa (×30,000). The scale bars represent 0.5 µm.

Scanning electron micrographs of treated and untreated *E. coli* are shown in Figure 3. No cell division was observed under any treatment conditions, which may be related to the activation time of the *E. coli* before LTHP treatment. Pressure treatment did not induce any additional modification of cellular size, and the target of the high pressure was mainly the damage to the cell wall or membrane of the microorganism [44]. The untreated *E. coli* appeared to be very plump, with no significant invaginations on the surface of the cell and only a few wrinkles. Freezing was able to make the surface of cell roughen (Figure 3B), but with such morphological changes it was difficult to directly cause their inactivation. Thus, the slight lethal effect observed with freezing was likely physiological. Low temperatures may cause the membranes to gel/stiffen (“solid”), depriving their biological function [45]. SEM revealed that pressure treatment at the 400 MPa level was sufficient to induce the occurrence of bud scars on the surfaces of the cells. Further, the cells appeared to be dehydrated, and deep folds could be clearly seen (Figure 3C). Although the changes in cell morphology were significant, it was uncertain whether the cell membrane or wall was disrupted (loss of membrane integrity). Tomasula et al. [41] concluded that such morphological changes were not lethal to the cells. Unlike the treated cells by high pressure at room temperatures, the LTHP-treated *E. coli* demonstrated no apparent invaginations. The cell membrane structure of *E. coli* was basically intact after LTHP treatment at −25 °C and 200 MPa, and a large number of pimples and swellings appeared on the surface (Figure 3D). When the pressure level was increased to 400 MPa (phase transition occurs), a gap of about a quarter of the surface area was clearly observed on the cell surface, and the integrity of the cell membrane was lost (Figure 3E). This damage can clearly be identified as lethal to the cell: the gap penetrated the cell membrane and wall, depriving their ability to insulate the internal and external environment. Two cycles of LTHP treatment at −25 °C and 400 MPa caused even the cytoplasm inside the cell to flow out, resulting in a significant reduction in cell size. Therefore, to some extent, it could be inferred that there were differences in the lethal mechanism between the pressure treatment under non-refrigerated room temperature conditions and LTHP phase transition conditions.

This was somewhat different from those reported in previous reports on the lethal mechanisms, which mainly focused on the disorganization among membrane phospholipids, changes in membrane potential, inactivation of metabolic enzymes, and destruction of nucleic acids, ribosome, and other cell structures [8,44,46,47,48]. They were obviously not as direct as membrane destruction, because it was extremely difficult to distinguish between lethal and sub-lethal cells through these factors [22]. In the case of obvious destructions of the cell membrane (wall), it was considered that there was no possibility for the cells to recover again. SEM images illustrated that there’s no distinct broken cell wall, not even during the maximum pressure of 400 MPa, which was consistent with previous studies [25,44]. However, Wang et al. [49] and Marx et al. [50] found that broken peptidoglycan layers and cell walls caused the cytoplasm to be exposed to the external environment, and inferred that these damages would cause the cells to be unable to recover later. This may be due to the difference in species; also, unlike them, the LTHP-treated cells did not fold or curl significantly when the phase transitions occurred, suggesting that the damages were most likely caused by a different form of force. The fluidity of the cell membrane decreases obviously at subzero temperatures, which makes the membrane gelatinize and become brittle [45], and the phase transition process will cause the destruction of the cell membrane. It must be noted that although high hydrostatic pressure treatments are considered to be isostatic, each cell has a different tolerance to pressure, meaning that even if the pressure treatment leads to total inactivation of the population, some individual cells may retain their morphological characteristics [44]. Hence, the picture did not fully represent the morphological state of all cells under the treatment condition. In order to strengthen the destructive effect caused by phase transitions, Edebo et al. [51] made the bacteria pass through the phase boundaries repeatedly by controlling the pressure, and found that the cell extract after treatment increased significantly. The volume variation is about 0.185 cm^3^/g for the phase boundary between Ice I to Ice III. Friction occurs inside the frozen milk and at the interface between the milk and cryogenic vial, along with a disintegration effect, which can be lethal to the cells. This could be used to explain why *E. coli* treated with LTHP shows a higher lethal levels rate than when treated under non refrigerated room temperature conditions.

**Figure 4:**
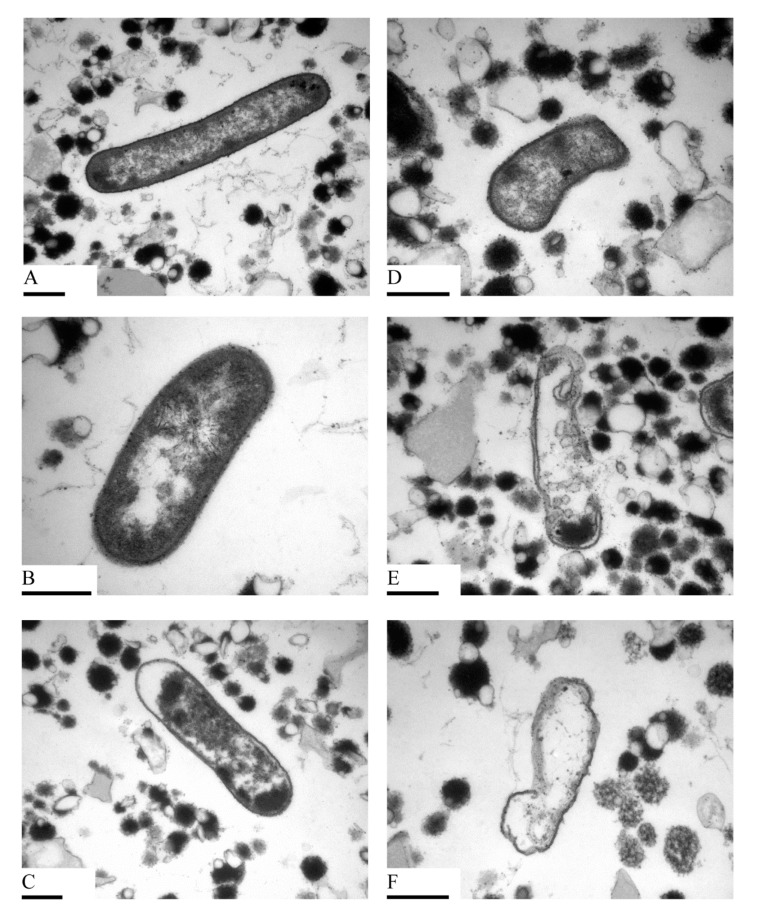
Transmission electron micrographs of *E. coli* resulting from different treatments: (**A**) control group (×40,000); (**B**) freezing (×80,000); (**C**) HP 400 MPa (×40,000); (**D**) LTHP 200 MPa (×60,000); (**E**) LTHP 400 MPa (×50,000); (**F**) LTHP two-cycle 400 MPa (×60,000). The scale bars represent 0.5 µm.

Although the cell wall or membrane is the main target of high-pressure treatment, intracellular injury should not be ignored [49]. Figure 4 shows the transmission electron micrographs of untreated and treated *E. coli*. The observed phenomena were similar to that of the scanning electron micrographs. Micrographs of SEM and TEM both provided evidence for the destruction of the *E. coli* membrane and wall structure by LTHP treatments. Untreated cells exhibited evenly distributed cytoplasm and an intact cell wall and cell membrane. A small number of electron-transparent regions were found in the frozen *E. coli* cells (Figure 4B). After treatment at 400 MPa, *E. coli* showed more electron permeable regions and partial cytoplasm aggregation (Figure 4C), similar to that observed by Wang et al. [49]. However, the cytoplasm bulging through small pores in the cell wall, observed in a previous study by Garcia-Gonzalez et al. [52], did not appear. In fact, it is still unclear whether the cytoplasm has been excluded from *E. coli* through this form, but the formation of such cytoplasmic clumps is obviously harmful to the cells. The TEM images demonstrated that the cells were folded to a small extent at −25 °C and 200 MPa (without phase transition), but it was obviously not enough to destroy the cell walls (Figure 4D). When the pressure exceeded 400 MPa (phase transition occurs), breakdown of the cell walls was evident, and the cytoplasm flowed out (Figure 4E). Therefore, it is plausible that the phase transitions destroy the cell wall and membrane, followed by losing the integrity of the cell structure, which is consistent with the observations of this study.

## 4. Conclusions

The obtained transient temperature–pressure data from frozen milk under pressure revealed that pressures over 300 MPa could induce an Ice I to Ice III phase transition. There was a significant difference in lethal levels of *E. coli* before and after the phase transition (at least 1.5 logarithms increase). Phase transition can enhance the lethal effect: the treatment at −25 °C and 300 MPa can achieve a lethal effect similar to the two-cycle treatment of 400 MPa at room temperature. In addition, injured cells were observed under all treatment conditions, and increasing the pressure cycles was able to transform injured cells into lethal cells. Micrographs of the TEM and SEM provided direct evidence for the breakdown of the cell membrane and cell wall by phase transitions. The designed special device provided the possibility of rapid cooling inside the high-pressure equipment to sterilize the microorganisms, but whether it can truly become a viable alternative non-thermal processing for the dairy industry may require further research on the repairing process of bacteria during storage.

In the present study, since the emphasis was to demonstrate the enhancement of microbial destruction under LTHP conditions, the process’s influence on quality was not included. It is expected that such an influence will be relatively small since it is truly a non-thermal process. However, since the freezing conditions could have some effects on proteins, future studies will involve targeted processes like pasteurization and their influence on the results.

## Figures and Tables

**Table 1:** The injury extent and lethality extent of the *E. coli* strains in inoculated milk resulting from different treatments. Mean values ± standard deviation (*n* = 3).

	Treatments		Lethality (log)	Injury (log)
Frozen or Not	Cycles	Pressure (MPa)		
Unfrozen	1	400	2.19 ± 0.13 ^a^	1.62 ± 0.29 ^a^
	2	400	2.85 ± 0.05 ^b^	1.60 ± 0.10 ^a^
Frozen	1	0.1	0.75 ± 0.03 ^c^	0.61 ± 0.18 ^b^
	1	100	1.33 ± 0.26 ^df^	0.96 ± 0.35 ^bc^
	1	200	1.30 ± 0.14 ^d^	0.85 ± 0.20 ^b^
	1	300	2.84 ± 0.25 ^b^	1.56 ± 0.33 ^ac^
	1	400	3.43 ± 0.10 ^e^	1.24 ± 0.24 ^abc^
	2	100	1.54 ± 0.13 ^df^	0.97 ± 0.37 ^bc^
	2	200	1.77 ± 0.35 ^f^	1.16 ± 0.26 ^abc^
	2	300	3.56 ± 0.03 ^e^	0.92 ± 0.26 ^b^
	2	400	4.53 ± 0.36 ^g^	0.72 ± 0.23 ^b^

In each column, different lowercase letters represent significant differences under different treatments, respectively (*p* < 0.05).
